# MX1: a bending-magnet crystallography beamline serving both chemical and macromolecular crystallography communities at the Australian Synchrotron

**DOI:** 10.1107/S1600577514021717

**Published:** 2015-01-01

**Authors:** Nathan Philip Cowieson, David Aragao, Mark Clift, Daniel J. Ericsson, Christine Gee, Stephen J. Harrop, Nathan Mudie, Santosh Panjikar, Jason R. Price, Alan Riboldi-Tunnicliffe, Rachel Williamson, Tom Caradoc-Davies

**Affiliations:** a MX Beamlines, Australian Synchrotron, 800 Blackburn Road, Clayton, Victoria 3168, Australia

**Keywords:** crystallography, beamline, bending magnet

## Abstract

MX1 is a bending-magnet crystallography beamline at the 3 GeV Australian Synchrotron. The beamline delivers hard X-rays in the energy range from 8 to 18 keV to a focal spot at the sample position of 120 µm FWHM. The beamline endstation and ancillary equipment facilitate local and remote access for both chemical and biological macromolecular crystallography. Here, the design of the beamline and endstation are discussed. The beamline has enjoyed a full user program for the last seven years and scientific highlights from the user program are also presented.

## Abstract 2

The macromolecular crystallography beamline MX1 at the Australian Synchrotron is described.

## 1. Introduction   

The macromolecular crystallography beamline (MX1) at the Australian Synchrotron is a general-purpose crystallography beamline, equipped to service the needs of the protein and chemical crystallography communities. The beamline has a bending-magnet source in the 3 GeV storage ring of the Australian Synchrotron. Two vertical focusing mirrors and a sagittally bent second crystal in the double-crystal monochromator deliver 3.3 × 10^11^ photons s^−1^ (Owen *et al.*, 2009 ▶) to a 120 µm-diameter (FWHM) circular spot at the sample position. Energy can be changed by a user within the range from 8 to 18 keV (see Table 1 ▶). The endstation features a sample-changing robot, κ goniometer and detector that can be moved around θ.

MX1 began hosting experiments in early 2007 and has run a fully subscribed user program to the present day. During this time the chemical crystallography community has made increasing use of the beamline with this community presently accounting for around 30% of the total available time. Depositions to the Protein Data Bank from the MX1 beamline have remained steady at around 50–60 per year since 2009.^1^

## 2. Beamline overview   

### 2.1. Beamline optics and hardware   

**Figure 1:**
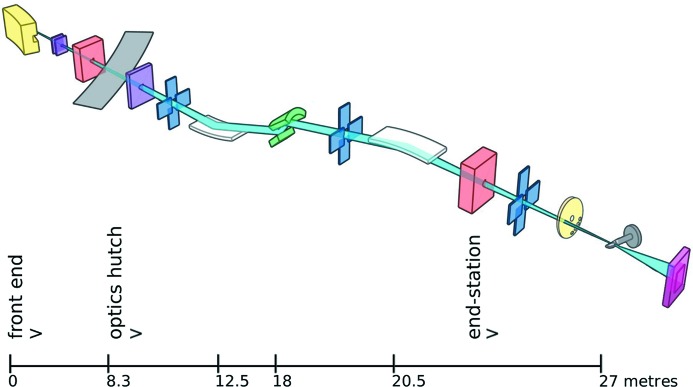
Schematic layout of the MX1 beamline. Components are bending-magnet source (yellow), beam-defining masks (purple), safety shutters (peach), storage-ring wall (light grey), slits (blue), mirrors (white), monochromator (green), filter wheel (yellow), goniometer (dark grey) and CCD detector (pink). Distances are metres from the source.

The optical layout of the MX1 beamline is shown schematically in Fig. 1 ▶. The source and optics deliver 3.3 × 10^11^ photons s^−1^ to a focused spot of 120 µm (FWHM and at 13 keV) at the sample position. Automated energy changes are available to users of the beamline in the range 8–18 keV.

Monochromation is achieved by a Si(111) double-crystal monochromator (DCM). The second crystal has a variable sagittal bend for horizontal focusing. The beamline has a horizontal acceptance of 1.7 mrad; this is limited by the area of cylindrical bend on the DCM second crystal. Beam outside of this acceptance area is rejected by a set of water-cooled white-beam slits directly upstream of the DCM.

Upstream of the white-beam slits the beam is vertically collimated by a bounce-up bent silicon mirror. A rubidium stripe on the mirror allows propagation of X-rays at higher energy while the silicon surface is used for harmonic rejection at lower energies (*i.e.* <7 keV). A second mirror with similar properties downstream of the DCM gives bounce-down vertical focusing towards the sample position.

### 2.2. Design and features of the goniometer, filter wheel and fast shutter   

Following the vertical focusing mirror (VFM) the beam passes through a final set of guard slits and through a beryllium window into a helium-purged assembly that houses the fast shutter and a filter wheel.

The filter wheel is an aluminium disk cut with a taper from 0.05 to 3 mm giving a continuous attenuation range from 0 to 60% transmission of the beam at 13 keV. A set of apertures in the filter wheel are either left open or covered by various thicknesses of aluminium foil giving an additional non-continuous set of attenuation options between 60 and 100%.

A fast rotary sample shutter is located 500 mm upstream of the sample. Beam steering and positional feedback are provided by optical visualization of the beam on a neodymium-doped yttrium aluminium garnet (YAG) crystal attached to the fast shutter (MacDowell *et al.*, 2004 ▶). A 6 mm-diameter stainless steel cylinder rotates to allow the beam to pass through a small slot cut through the diameter. When the shutter closes, a small piece of YAG rotates into the path of the beam and a CCD camera (Point Grey) records an image of the beam fluorescence on the shutter YAG. The centroid of the beam image is calculated from CCD camera input using *areaDetector* (Rivers, 2010 ▶). The CCD reads out at 15 frames s^−1^ and *areaDetector* calculates centroids from a rolling eight-frame box-car average that also reads out at 15 frames s^−1^. The beam is steered to the reference position by an EPICS PID feedback loop that samples the beam image centroids every 0.5 s using piezo motors on the DCM second-crystal roll for horizontal steering and on the VFM for vertical steering. The timing of the feedback loop is limited by the EPICS PID loop at 0.5 s and the accuracy is limited by the fitting of a Gaussian to the beam image; this is better that 1 µm for a well focused beam.

The goniometer rotates the sample around a horizontal axis and data are collected in reversed ϕ mode. Two precision linear motors (PI) are used to centre the sample on the rotation axis with a sphere of confusion better than 2 µm.

### 2.3. Experimental parameters   

The size of the beam at the sample position at MX1 is 120 µm in diameter (FWHM). In general, the beam size allows for routine collection of data from crystals with dimensions larger than around 50 µm for protein samples but has achieved good results for chemical crystals smaller than 10 µm with strongly scattering elements. Typically a diffraction image is collected in 1.0 s with a beam attenuated to around 20% transmission. There is dead-time of approximately 1.0 s per image allowing for a 180 image dataset to be collected in around 6 min. The SSRL Automated Mounting (SAM) type sample-mounting robot (Cohen *et al.*, 2002 ▶) can exchange samples in approximately 2 min (not including time for sample centring or data collection). MX1 uses the *Blu-Ice* control system (McPhillips *et al.*, 2002 ▶), and with ‘click-to-centre’ sample centring a newly mounted crystal can be centred for data collection in around 30 s.

**Figure 2:**
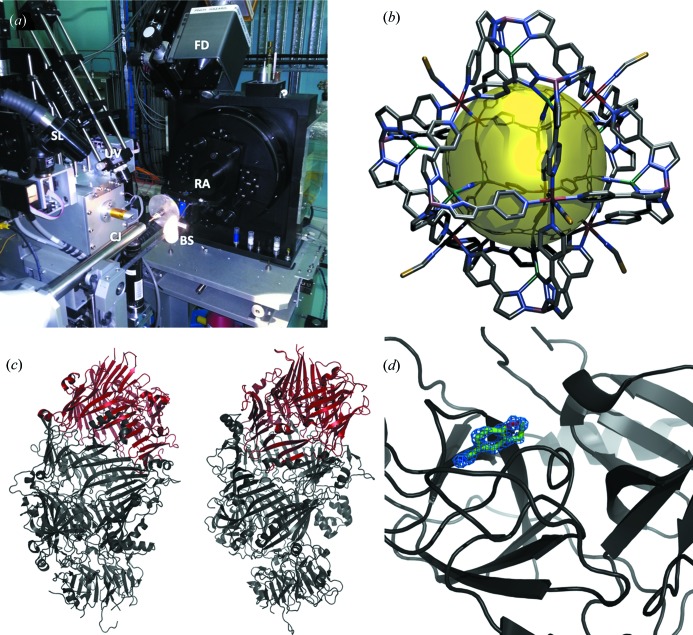
(*a*) MX1 sample environment showing the fluorescence detector (FD), the rotation axis (RA), the cryojet (CJ) and illuminated back-stop (BS) projecting in from the bottom left, the sample light projecting in from the left middle (SL), and the UV laser for radiation-induced phasing (UV) projecting in from the top left. (*b*) Stick representation of a supramolecular assembly (Duriska *et al.*, 2009*a* ▶,*b* ▶). The gold sphere is used to illustrate the void space and does not represent a real feature. (*c*) Orthogonal views of the B:C component complex (4IGL) of the bacterial ABC toxin. The C component is shown in red and the B component in grey. (*d*) View of a fragment bound to bovine trypsin (4AB9). Electron density around the fragment is show contoured at 1σ.

The beam exits the photon-delivery system through a small aperture cut through a mirror that is mounted on the collimator. The mirror is at 45° to the beam direction and is viewed by a camera that is mounted perpendicular to the beam (Fig. 2*a* ▶). The camera and mirror give a view of the sample along the axis of the beam with a fixed five-fold magnification. A cold sample light is focused on a disk of thin white packing foam that is mounted on the beam stop. The foam is transparent to X-rays and allows the sample to be back-lit without the need for moving a light source out of the direct beam each time a diffraction image is measured (MacDowell *et al.*, 2004 ▶). The foam back-light allows an optical view of the sample to be continuously available during data collection and allows a matching optical still image of the sample to be saved along with each diffraction image in the normal operating mode of the beamline.

A Quantum 210r CCD detector (ADSC) is mounted on an A-frame allowing sample-to-detector distances between 72 and 800 mm. The frame also supports movement of the detector around 2θ.

Samples can be manually mounted or mounted *via* a SAM system that allows for remote data collection (Cohen *et al.*, 2002 ▶).

A CryoJet 5 (Oxford Instruments) maintains the temperature of the sample.

The beamline is extremely stable (merging *R* in the lowest resolution bin is typically less than 2% and often close to 1%) and is therefore a powerful tool for anomalous phasing. Energy can be readily changed by the users in the range from 8 to 18 keV and with staff intervention can be set up as low as 6 keV. This range covers most of the useful absorption edges with the lower energies being particularly useful for sulfur SAD phasing. A sensitive silicon-drift fluorescence detector (Vortex 90EX, Hitachi) is automatically moved to an optimal position a few millimetres from the sample (*via* a rodless pneumatic actuator) for MAD and excitation scans. X-ray attenuation is optimized by a routine within the *Blu-Ice* control system to avoid count overload on the fluorescence detector. Finally a UV laser can be used for radiation-damage-induced phasing (de Sanctis *et al.*, 2011 ▶; Nanao & Ravelli, 2006 ▶) (Fig. 2*a* ▶).

The MX1 beamline features automated data processing. Collection of a single diffraction image triggers indexing by the program *LabelIt* (Sauter *et al.*, 2004 ▶). Collection of a dataset triggers automated indexing and full integration and scaling using *xdsme* (Legrand, undated ▶) and *Aimless* (Evans & Murshudov, 2013 ▶). *xdsme* is a Python wrapper that makes use of the programs *XDS* (Kabsch, 2010 ▶) and *Pointless* (Evans, 2006 ▶). Statistical descriptors of the data are harvested from the processed data and displayed by a web client at the beamlines in close to real time. The system is useful in guiding the strategic decisions made by a user of the beamline.

Data are stored live to two independent data storage facilities for redundancy, backup and remote user access. A local four-node EMC Isilon IBM-based SANS with a total capacity of 520 Tb stores a compressed single file copy in SquashFS format of the whole experiment while a second copy is transferred offsite with both raw data and autoprocessing metadata stored in *MyTardis* (Androulakis *et al.*, 2008 ▶).^2^ For opt-in user authorized datasets, raw data are made publicly available for the world-wide community *via* the same *MyTardis* system.

### 2.4. Ancillary facilities   

The beamline is supported by a PC2 certified biochemistry laboratory with fume hoods, pH meters, centrifuges, balances and other common laboratory equipment. A selection of heavy atom and halide salts and a Xenon Chamber (Hampton Research) are available for derivatization of crystals at the beamline. Xenon derivatization should be arranged in advance.

## 3. Facility access   

Access to the Australian Synchrotron is by a merit-based scheme. Regular users access the beamlines *via* a collaborative access program (CAP) whereby several laboratories group together to submit a single application for beam time that will cover a whole year. Researchers who use the beamlines less frequently can apply for rapid access time and these applications can be submitted throughout the year. All applications are independently peer-reviewed and scored. Experiments on the MX1 beamline can be conducted remotely.

## 4. Highlights   

Much of the success of the MX1 beamline can be attributed to an excellent body of both chemical and protein crystallography users. The beamline has strong output of high-impact science for both of these disciplines.

### 4.1. Chemical crystallography   

There has been widespread interest in multifunctional materials from supramolecular assemblies where simple subunits combine *via* self-assembly to yield large molecular compounds with complex emergent properties. Often the structural analysis of these large assemblies required the use of synchrotron radiation over a laboratory source, due to weak diffraction and/or poor signal to noise due to large domains of disordered solvent and/or small crystals. One area of interest is the use of crystalline materials that possess large void spaces to allow sorption/desorption of gases and liquids. Structural characterization was carried out at the high-throughput MX1 beamline of discrete supramolecular ‘nanoball’ cages (around 3 nm across) (Duriska *et al.*, 2009*a* ▶,*b* ▶) (Fig. 2*b* ▶). The Fe^2+^ complex shows spin crossover and can be activated by temperature, laser irradiation [light-induced spin state trapping (LIESST)] and guest perturbation.

### 4.2. Protein crystallography   

#### 4.2.1. B:C component of bacterial ABC toxin   

A recent highlight in the area of protein crystallography was the determination of the structure of the B:C component of a bacterial ABC-class toxin (Busby *et al.*, 2013 ▶) (Fig. 2*c* ▶). This class of toxin has potential as an insecticidal agent and also implications for human disease. The structure gives insight into the different roles that the various components of this complex play and provides an archetype structure for a domain family.

A high-resolution native dataset was collected on the MX2 micro-focus beamline at the Australian Synchrotron while phasing of the data was *via* four selenium SAD datasets and a tantalum bromide derivative. The paper illustrates the utility of the MX1 beamline for phasing challenging structures.

#### 4.2.2. A training dataset for structure-based drug design   

One of the largest projects attempted at MX1 was the creation of a large body of data using crystallography, isothermal calorimetry and surface plasmon resonance to characterize the interactions of molecules from a library of compounds with bovine trypsin (Newman *et al.*, 2012 ▶) (Fig. 2*d* ▶). The result was used as a training dataset for a computational drug design competition.

The project involved collection of more than 1000 diffraction datasets from trypsin. Robotic sample loading and automated data reduction and structure solution software running at the beamline were critical to the feasibility and ultimate success of the project. The study demonstrates that high-throughput crystallography projects can be undertaken on the MX1 beamline.

## 5. Discussion and conclusions   

The MX1 beamline enjoys use by both the protein and chemical crystallography communities within Australia. This mixture has led to improvements on the beamline, diversification of the experience of the beamline staff and ultimately benefited both user communities.

The stability and ease of use of this bending-magnet beamline make it particularly good for high-throughput crystallography projects such as fragment screening. High data quality, user-controlled energy changes, and ancillary equipment such as the UV laser make the beamline an excellent tool for phasing difficult structures.

## Figures and Tables

**Table 1:** Beamline details

Beamline name	Macro Crystallography MX1
Source type	Bending magnet
Monochromator	Double-crystal Si(111) water-cooled
Energy range	718keV user controlled
Wavelength range	1.770.69
Mirrors	Two Si 1300mm 110mm with 50mm Rh stripes; one bounce-up collimating, one bounce-down focusing
Beam size, uncollimated (FWHM H V)	120m 120m
Photon flux	3.6 10^11^photonss^1^ at 13keV
Goniometer	Horizontal air-bearing
Cryo capability	CryoJet 5 (Oxford Instruments, temperature range 85 and 500K)
CCD detector	Quantum 210r (ADSC)
Fluorescence detector	Vortex Si-drift detector (Hitachi)
Sample mounting	SAM system (Cohen *et al.*, 2002 ▶) supports use of the SSRL style cassettes or ALS style Uni-pucks.

## Footnotes

1 Data retrieved on 12 February 2013 from Biosync: http://biosync.sbkb.org/stats.do?stats_sec=RGNL&stats_focus_lvl=SITE&stats_site=AUSTRALIAN%20SYNCHROTRON.

2 Retrieved on 12 February 2013 from *Store.Synchrotron*: https://store.synchrotron.org.au/.
